# Developmental Dynamics of Post-Selection Thymic DN iNKT

**DOI:** 10.1371/journal.pone.0043509

**Published:** 2012-08-22

**Authors:** Maryam Yassai, Brian Cooley, Jack Gorski

**Affiliations:** 1 Blood Research Institute, BloodCenter of Wisconsin, Milwaukee, Wisconsin, United States of America; 2 Department of Orthopedic Surgery, Medical College of Wisconsin, Milwaukee, Wisconsin, United States of America; 3 Department of Microbiology and Molecular Genetics, Medical College of Wisconsin, Milwaukee, Wisconsin, United States of America; Karolinska Institutet, Sweden

## Abstract

**Background:**

Invariant natural killer T (iNKT) cells develop in the thymus and branch off from the maturation pathway of conventional T cell at the DP stage. While different stages of iNKT cellular development have been defined, the actual time that iNKT cell precursors spend at each stage is still unknown.

**Methodology/Principal Finding:**

Here we report on maturation dynamics of post-selection DN iNKT cells by injecting wild-type DP^dim^ thymocytes into the thymus of TCRα^−/−^ mice and using the Vα14-Jα18 rearrangements as a molecular marker to follow the maturation dynamics of these cells.

**Conclusion/Significance:**

This study shows that the developmental dynamics of DN iNKT cells in DP^dim^ are very rapid and that it takes less than 1 day to down-regulate CD4 and CD8 and become DN. These DN cells are precursors of peripheral DN iNKT cells and appear in the spleen in 1–2 days. Thymic DN iNKT residents are predominantly derived from cells that quickly return from the periphery. The expansion of a very small subset of DN iNKT precursors could also play a small role in this process. These data are an example of measuring T cell maturation in the thymus and show that the maturation dynamics of selected DN iNKT cells fall within the same general time frame as conventional αβ T cells.

## Introduction

T cells are divided into several subsets based on the expression of their antigen receptor and their function. T cells expressing the αβ T cell receptor (TCR) constitute the majority of peripheral T cells, and they generally respond to peptides presented by MHC class I or class II molecules. A small subset of αβ T cells respond to lipids presented by a non-classical MHC molecule, CD1d. Because they also express surface markers normally associated with NK cells, they are referred to as NKT cells. αβ T cells develop in the thymus from early thymic progenitors that are released from the bone marrow and colonize the thymus. Different stages of development are defined by the expression of T cell receptor (TCR), its co-receptors, CD4 and CD8, and other accessory molecules. TCR expression requires rearrangement of different gene segments: variable (V), diversity (D), and joining (J). The first gene to rearrange is the TCRβ chain gene. Because the rearrangement process is imprecise, two thirds of rearrangements are out-of-frame and thus non- functional. Selection of the cells that have in-frame rearrangement is referred to as β selection. This selection takes place in cells that do not yet express CD4 and/or CD8 co-receptors (CD4^−^CD8^−^ double-negative cells, DN). DN thymocytes can be further divided into stages defined by expression of CD44 and CD25: CD44^+^CD25^−^ (DN1), CD44^+^CD25^+^ (DN2), CD44^−^CD25^+^ (DN3), and CD44^−^CD25^−^ (DN4) [1]. TCR β rearrangement takes place at DN2, and β selection happens at the DN3 stage. TCR β selection is followed by up-regulation of CD4 and CD8, which leads to the next developmental stage, CD4^+^CD8^+^ double-positive cells (DP). TCRα rearrangement takes place at the DP stage, and thymocytes that have productively rearranged TCRα will then be subjected to a series of selection events, referred to as positive and negative selection. As a result of these selection processes, thymocytes that can bind to MHC molecules and are non-reactive to self-antigens are selected for further development. The majority of the positively selected DP cells become CD8 single positive (SP8) or CD4 single positive (SP4) by down-regulating either CD4 or CD8 co-receptors. Single positive thymocytes will undergo additional negative selection and exit the thymus.

It has been shown that iNKT cells and conventional T cells originate from a common pool of DP precursors [2]–[5], and DP^dim^ thymocytes contain iNKT precursors that had already undergone selection [2]. Murine iNKT cells are CD4^+^ or CD4^−^CD8^−^ (DN), these cells express an invariant TCRα chain (Vα14-Jα18) [6] that is paired with a limited number of β chains (Vβ8.2, Vβ7, or Vβ2) [7]. iNKT cells express a memory or activated phenotype [8]. It has been shown that thymic precursors of iNKT cells express CD44 [9], [10] which is a marker normally associated with T cell memory [11]. iNKT cells exit the thymus as immature NK1.1^−^ cells, complete their maturation and express NK1.1 in the periphery [9], [12], [13]. iNKT cells that reside in the thymus are mature, express a high level of CD44, and are NK1.1^+^ [14].

Although we understand the general stages of iNKT cell development [15]–[17], the actual dynamics of their maturation is still unknown. Here we report on the dynamics of DN iNKT cell development from DP^dim^ precursors. DP^dim^ is an interesting population because it consists of cells that are gaining expression of CD4 and CD8 (DN to DP), and cells that are losing expression of CD4 and CD8 (DP to DN). We show that CD3-expressing DP^dim^ thymocytes are enriched in iNKT precursors. To measure the dynamics of iNKT maturation after thymic injection of DP^dim^ cells, we identified iNKT precursors or mature cells by testing for the iNKT-specific Vα14-Jα18 rearrangement (iNKT rearrangement). Injecting cells into the thymic lobes has been used for studying the developmental potential of bone marrow cells [18], thymocyte subsets [19], [20], and iNKT cells [5], [13], [14]. In this study, wild-type DP^dim^ or DN thymocytes were injected into the thymus of TCRα^−/−^ mice, and the developmental dynamics of iNKT cells were studied.

## Results

### Rearrangement Analysis of DP^dim^

**Figure 1:**
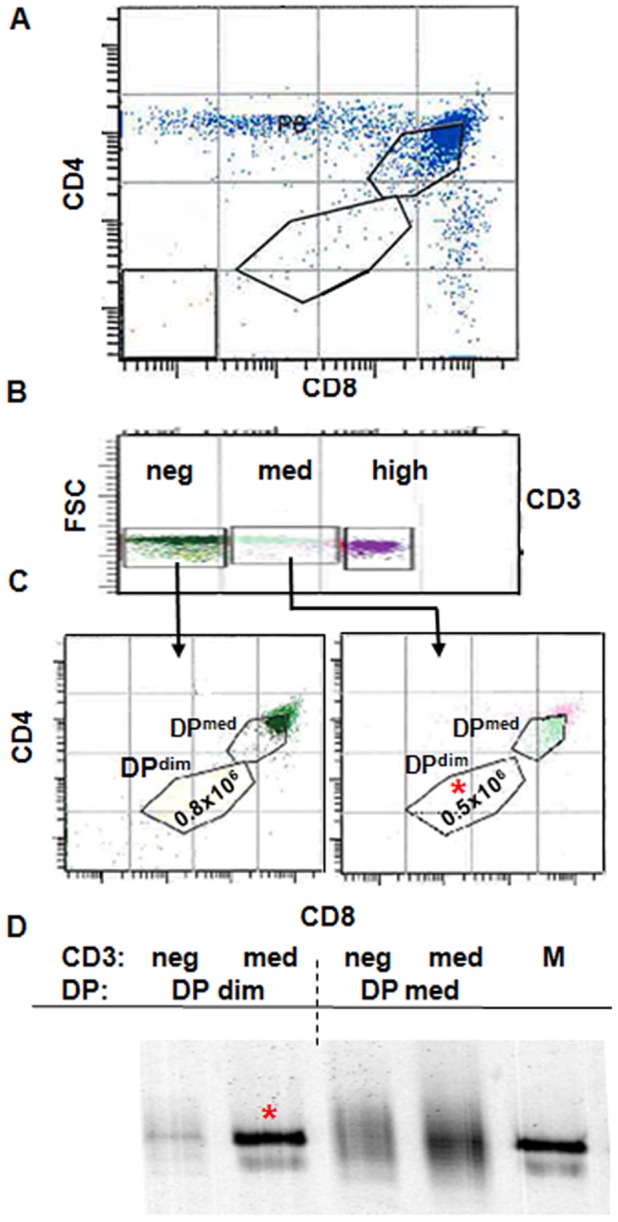
Thymocytes with iNKT rearrangements are found in the DP^dim^ population. A. FACS profile of thymocytes based on CD4 and CD8 expression and defined gates for DP^dim^ and DP^med^ thymocytes. B. FACS profile of thymocytes based on CD3 expression and defined gates for CD3^neg^, CD3^med^ and CD3^hi^. C. FACS profile of thymocytes based on CD4 and CD8 expression through CD3^neg^ and CD3^med^ populations. Numbers inside DP^dim^ gates represent the number of collected cells. D. Rearrangement analysis of Vα14-Jα18 in different sorted DP populations. The asterisk shows the population (CD3^med^ DP^dim^) in which iNKT cells are observed, and lane “M” represents the iNKT rearrangement in the spleen of wild type C57BL/6 mouse and serves as size marker.

The DP^dim^ population is a small component of normal thymi and is thought to represent a transitional stage between DN and DP thymocytes (Figure 1A). DP^dim^ thymocytes can be DN cells that have just passed TCRβ selection, have started to up-regulate CD4 and CD8, and are still CD3^neg^. We refer to these as DP^dim1^. DP thymocytes that have rearranged TCRα, generated paired αβ TCR, passed positive selection, have no requirement for either CD4 or CD8 co-receptors, and have started to down-regulate CD4 and CD8 expression, would also be DP^dim^. We refer to these as DP^dim2^. Because DP^dim2^ have successfully rearranged the TCR, they express CD3, which can be used to differentiate the two DP^dim^ populations. To test this interpretation, we sorted thymocytes on the basis of CD3 expression (Figure 1B). The CD3^neg^ or CD3^med^ cells were then gated on the DP^dim^ cells and as well as DP cells that expressed intermediate levels of CD4 and CD8 (DP^med^) (Figure 1C). Rearrangement analysis showed that iNKT precursors, as defined by the Vα14-Jα18 rearrangement, are highly enriched in the CD3^med^ DP^dim^ fraction (Figure 1D). The faint band in the CD3^neg^ DP^dim^ lane may represent a small proportion of iNKT precursors that have down-regulated CD3. The DP^med^ analysis shows rearrangements with single base pair spacing due to out-of-frame rearrangements (Figure 1D, right lanes). The decreased intensity in CD3^neg^ DP^dim^ cells is most likely due to the fact that they have just started to rearrange the α**-**chain. In sorting for the injection experiments we chose not to use anti-CD3 antibodies to avoid possible TCR stimulation, and thus only transferred DP^dim^ cells.

### iNKT Cells can be Detected in DN Thymocytes Following DP^dim^ Injection

**Figure 2:**
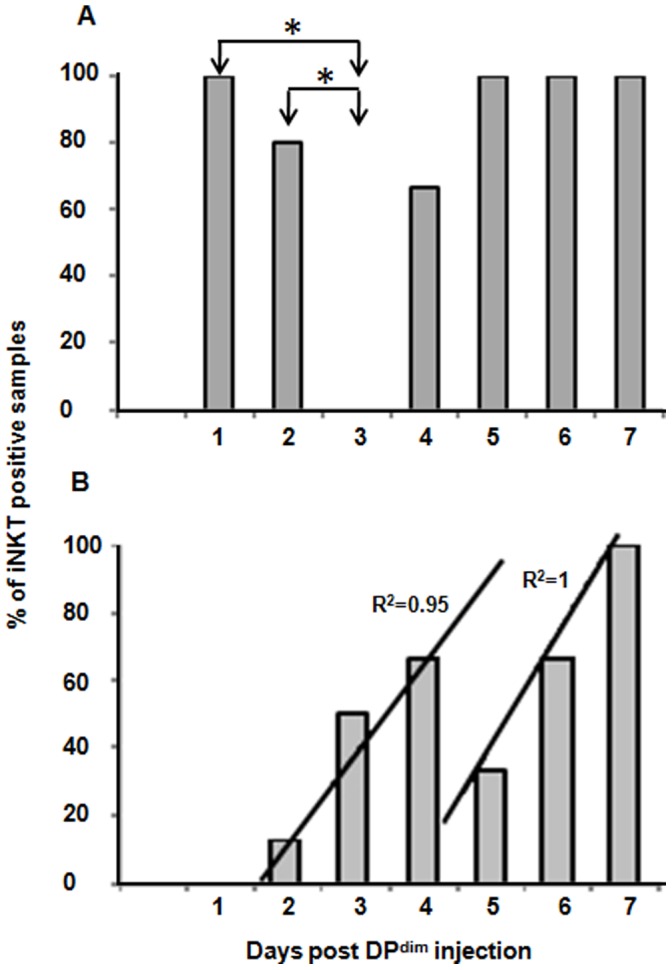
iNKT rearrangements in TCRα^−/−^ mice after intrathymic injection of DP^dim^ cells. The *X axis* shows days post injection, and the *Y axis* shows the percent of mice analyzed that had iNKT rearrangements. A. DN thymocytes were analyzed. The arrows and asterisks indicate statistically significant changes. Fisher’s exact test was used to compute the P value from contingency table (day 1& 3, P = 0.04; day 2&3, P = 0.03). B. Total splenocytes were analyzed. The correlation between the two linear regression lines was calculated based on the Zar’s method (P = 0.008). The actual data for each time point are in Table 1.

In seven independent experiments DP^dim^ thymocytes were collected and injected intrathymically into 29 TCRα^−/−^ mice. Thymi were collected at different times post injection, and DN thymocytes were sorted from 24 of these mice and analyzed for iNKT rearrangements. The percentage of mice that were positive for the iNKT rearrangement at each day is shown in Figure 2A and Table 1. The results show that the iNKT precursors in the injected DP^dim^ population lose the expression of CD4 and CD8 and become DN iNKT thymocyte as early as 18 hours post injection (three out of three mice in three different experiments). On day 3 there were no mice with the iNKT rearrangement in the DN compartment. This change in the number of mice with iNKT rearrangements at day 3 was statistically significant when compared with day 1 and day 2 using a 2×2 contingency analysis (Pavg = 0.035). At day 4 iNKT were detected in two of three mice and from then on, all the mice examined had DN cells with iNKT rearrangement signatures. Thus, while the transition from DP^dim^ to DN iNKT is rapid, the DN iNKT population is not stable and leaves the thymus by day 3. The DN thymic compartment is repopulated by iNKT after day 3.

**Table 1:** iNKT rearrangement in DN thymocytes and spleen samples obtained from TCRα^−/−^ mice at different days post DP^dim^ injection.

Days Post DP^dim^ Injection	0	1	2	3	4	5	6	7
DN Thymocytes Analyzed^a^	3	3	5	4	3	3	4	2
% iNKT Positive DNs	0	100	80	0	66.6	100	100	100
Spleen Samples Analyzed	3	3	8	4	3	3	6	2
% iNKT Positive Spleens	0	0	12.5	50	66.6	33.3	66.6	100

a Data from 7 experiments are summarized.

As would be expected, iNKT rearrangement was not observed in DN thymocytes of animals that did not receive DP^dim^ cells (day 0). The gel rearrangement analyses contained a size marker and a control PCR (water only). An example of results from such an experiment is shown in Figure S1.

### iNKT Cells can be Detected in the Spleen Following DP^dim^ Injection

Splenocytes were collected from the same 29 mice and analyzed for iNKT rearrangement. The percentage of mice with positive signals for iNKT rearrangement is shown in Figure 2B and Table 1. The results show that iNKT can be detected in the spleens of some mice as early as 2 days after injection and that the number increases at day 3 and day 4 (50% and 66.7%, respectively). There was a decrease in the percentage of animals that had positive signal at day 5 (33.3%), after which the number increased again (day 6 and day 7). iNKT cells arriving in the spleen at days 2, 3, and 4 had a very similar rate as those iNKT cells arriving at days 5, 6, and 7. Comparison of the two regression lines showed that the difference between the slopes of the lines is not statistically significant (P = 0.41) and can be considered the same. The difference between the intercepts is statistically significant (P = 0.008) and the lines can be considered parallel.

Overall, the migration of iNKT cell precursors from DP^dim^ to the spleen requires 2–3 days. As would be expected, iNKT rearrangement was not observed in the spleens of animals that did not receive DP^dim^ cells (day 0).

To ensure that the fragment generated in the PCR using Vα14 and Jα18 primers represents the iNKT rearrangement, the PCR products generated from the DNA from DN thymocytes and total splenocytes collected at day 4 post injection of DP^dim^ were subjected to confirmatory sequencing. The result showed only one N-nucleotide at the Vα-Jα junction (Figure S2) that is in keeping with published data [6].

### Thymic DN iNKT Cells are Precursors of Splenic iNKT Cells

The times of arrival of iNKT in the DN compartment and in the spleen is compatible with passage of iNKT precursors through the thymic DN stage prior to exit to the spleen. The appearance of splenic iNKT cell on days 2, and 3 coincides with the loss of DN iNKT thymocytes generated from the injected DP^dim^ (days 1 & 2), which is in keeping with a precursor-product relationship.

To directly test the precursor-product relationship of thymic DN iNKT with splenic iNKT cells, we injected wild-type DN iNKT cells into the thymi of four TCRα^−/−^ mice and spleen of recipient mice were checked for iNKT rearrangement at 1, 2, 3, and 4 days after injection. iNKT rearrangement was found in the spleen 1 day post injection, indicating that DN iNKT can give rise to splenic iNKT (data not shown).

### DN iNKT Cells can Return to the Thymus

**Figure 3:**
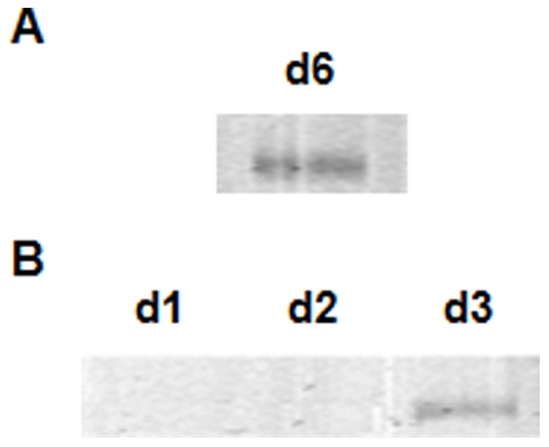
DN iNKT cells can return to the thymus. DP^dim^ thymocytes were injected in the thymi of four TCR Cα^−/−^ mice and 6 days post transfer, the thymi were pooled and DN thymocytes were sorted. A.iNKT rearrangement from DN thymocytes 6 days post transfer of DP^dim^. The newly generated DN day 6 thymocytes were transferred to the periphery of TCR Cα^−/−^ mice. B. iNKT rearrangements in the thymus at different days post peripheral transfer of day 6 DN thymocytes.

The data in Figure 2A posed the question as to the origin of the repopulated cells (day 4). One possibility is the return of iNKT cells from the periphery. To investigate this possibility we injected DP^dim^ in the thymi of four TCR Cα^−/−^ mice. Six days post transfer, the thymi were pooled and DN thymocytes were sorted. As expected these DN thymocytes contained iNKT cells (Figure 3A). The newly generated DN thymocytes were then injected in the periphery of TCR Cα^−/−^ mice and iNKT rearrangement was analyzed in the thymus at days one to three post intravenous injection (Figure 3B). The results showed that 3 days after intravenous injection of the newly generated DN iNKT cells, they could be detected in the thymus. Due to the low number of day 6 thymic DN iNKT cells among total DN cells that were injected, we were not able to detect them at earlier time points.

### DN iNKT Cells Generated from DP^dim^ Injection Express CD44, a Memory or Activated Phenotype

**Figure 4:**
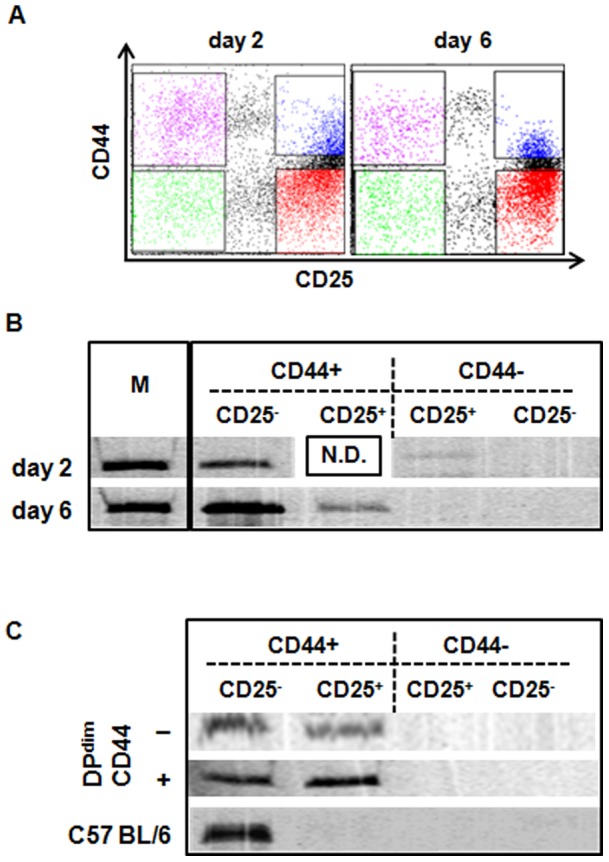
DN iNKT cells that are generated from DP^dim^ injection express CD44. A. FACS profile of DN thymocytes based on CD44 and CD25 expression for thymocytes collected at day 2 and day 6 post DP^dim^ injection. The sort gates for the four populations are shown. B. iNKT rearrangement in DN thymocytes from the four gated populations at day 2 and day 6, post DP^dim^ injection. “N.D.” not done due to the low number of CD44^+^ CD25^+^ cells on day 2. Lane “M” represents the iNKT rearrangement in the spleen of wild-type C57BL/6 mouse and serves as size marker. C. iNKT rearrangement analysis in the DN thymocyte subsets 18 hours after thymic injection of sorted CD44^−^ (Top row), and CD44^+^ (middle row) DP^dim^ cells. The bottom row shows iNKT rearrangement in DN thymocyte subsets from a C57BL/6 mouse representing the steady state pattern.

It has been shown that iNKT cells express a memory or activated phenotype based on CD44 expression [9], [10]. To test if DN iNKT cells generated from DP^dim^ injection have the same phenotype, we fractionated DN thymocytes generated at days 2 and 6 post DP^dim^ injection on the basis of CD44 and CD25 expression (Figure 4A). These time points were chosen because they represent the two phases of DN iNKT appearance. iNKT rearrangement analysis showed a strong signal in the DN1 (CD44^+^ CD25^−^) compartment at both time points (Figure 4B). In day two there were insufficient CD44^+^ CD25^+^ cells to perform analysis (Figure 4A, left panel). The faint band in CD44^−^ CD25^+^ is not aligned with the marker (Figure 4B).

We also investigated whether DP^dim^ iNKT precursors that did not express CD44 can acquire its expression. Therefore we fractionated DP^dim^ on the basis of CD44 expression and analyzed iNKT rearrangement in DN thymocytes 18 hours after the injection. Interestingly both the CD44^−^ and CD44^+^ subsets of DP^dim^ cells could give rise to DN iNKT cells (Figure 4C). Of further interest, at this early time there was a considerable number of CD44^+^ DN iNKT that were still expressing CD25. In a separate experiment we found that 26.8% of CD44^+^DP^dim^ and 9.4% of CD44^−^DP^dim^ expressed CD25 (data not shown). Therefore iNKT precursors that have matured to the DN stage still express CD25 for a period of time. In contrast to the early developmental stages the steady state distribution of iNKT does not include CD25 expressing cells.

### iNKT Lineages Generated from DP^dim^ can be Long Lived

**Figure 5:**
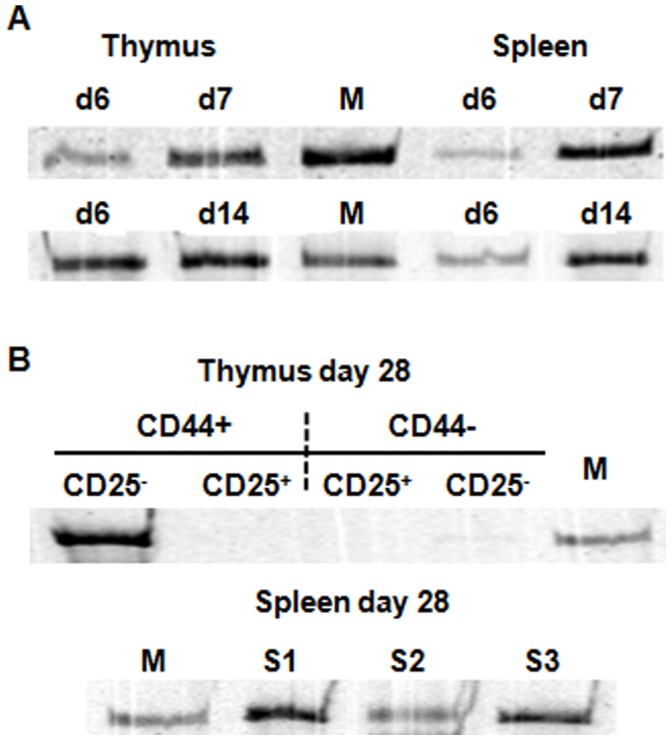
iNKT lineages generated from wild type DP^dim^ injection in TCRα^−/−^ mice can be long lived. A. iNKT arrangement in DN thymocytes and in splenocytes at day 6, day 7, and day 14 post DP^dim^ injection. Top and bottom rows represent separate experiments. The middle column “M” shows iNKT rearrangement as a marker. The dates post injection of wild type DP^dim^ are shown at the top of each band. B. iNKT rearrangement in thymus and spleen 28 days post DP^dim^ injection. Thymocytes from three TCRα^−/−^ mice were pooled at day 28 post injection, and DN thymocytes were sorted based on the expression of CD44 and CD25 (first row). At this time newly generated iNKTs are predominantly CD44^+^CD25^−^ with a small population that is CD44^−^CD25^−^. The second row shows the iNKT rearrangement in the spleens of the same three mice.

We have already shown that iNKT cells are present in the spleen and thymus at day 7 post injection. To determine the longevity of the iNKT lineages in these tissues, two independent experiments were performed in which thymus and spleen of the recipient mice were analyzed for the presence of iNKT rearrangement, at days 14 and 28 post DP^dim^ injection. iNKT rearrangements were detected in all spleen and un-fractionated DN thymus samples (Figure 5A). In the day 28 analysis, the DN thymocytes were further fractionated on the basis of CD44 and CD25expression. iNKT rearrangement was detected in all spleen samples, the majority of thymic iNKT cells were detected in CD44^+^ CD25^−^ fraction of DN thymocytes (Figure 5B). The faint band in CD44^−^ CD25^−^ fraction of DN thymocytes is due to T cells that happens to have iNKT rearrangement and are down regulating all surface markers. Also all three animals showed iNKT rearrangement in their spleen.

## Discussion

In this study we used rearrangement analysis together with intrathymic transfer of wild-type DP^dim^ to TCRα ^−/−^ mice to describe the dynamics of DN iNKT maturation in the absence of continuing input from the bone marrow. Since we had previously shown that rearrangement studies are sensitive techniques for studying thymocyte development [21], [22], we took advantage of the unique Vα14-Jα18 rearrangement and used it as a molecular marker for tracking the maturation of iNKT cells. The data showed a direct connection between DP^dim^ and DN iNKT cells. iNKT cells were detected as DN iNKT cells within 18 hours post DP^dim^ transfer. It is interesting that the maturation dynamics of DP^dim^ iNKT precursors occur rapidly with down-regulation of CD4 and CD8 receptors in 18 hours. The DN iNKT cells remained in the thymus for two days and were gone by day 3. The disappearance of DN iNKT cells from the thymus and their appearance in the spleen implies a precursor-product relationship. This relationship was confirmed by injecting wild-type DN thymocytes into TCRα^−/−^ thymi and detecting iNKT cells in the spleen after 18 hours.

It should be pointed out that in our experimental procedures the number of cells recovered from a day of sorting limits the number of recipient mice that can be analyzed. The relation between time and developmental pattern is inferential because each mouse represents an independent maturation sequence, and the timing of maturation may not be identical in all animals. Reproducibility can be indirectly estimated by analyzing the outcomes of experiments when two or more mice from the same transfer are analyzed on the same day. This occurred in experiments where thymocytes were pooled from multiple mice for sorting. The 16 individual spleen samples showed the same result 75% of the time.

**Figure 6:**
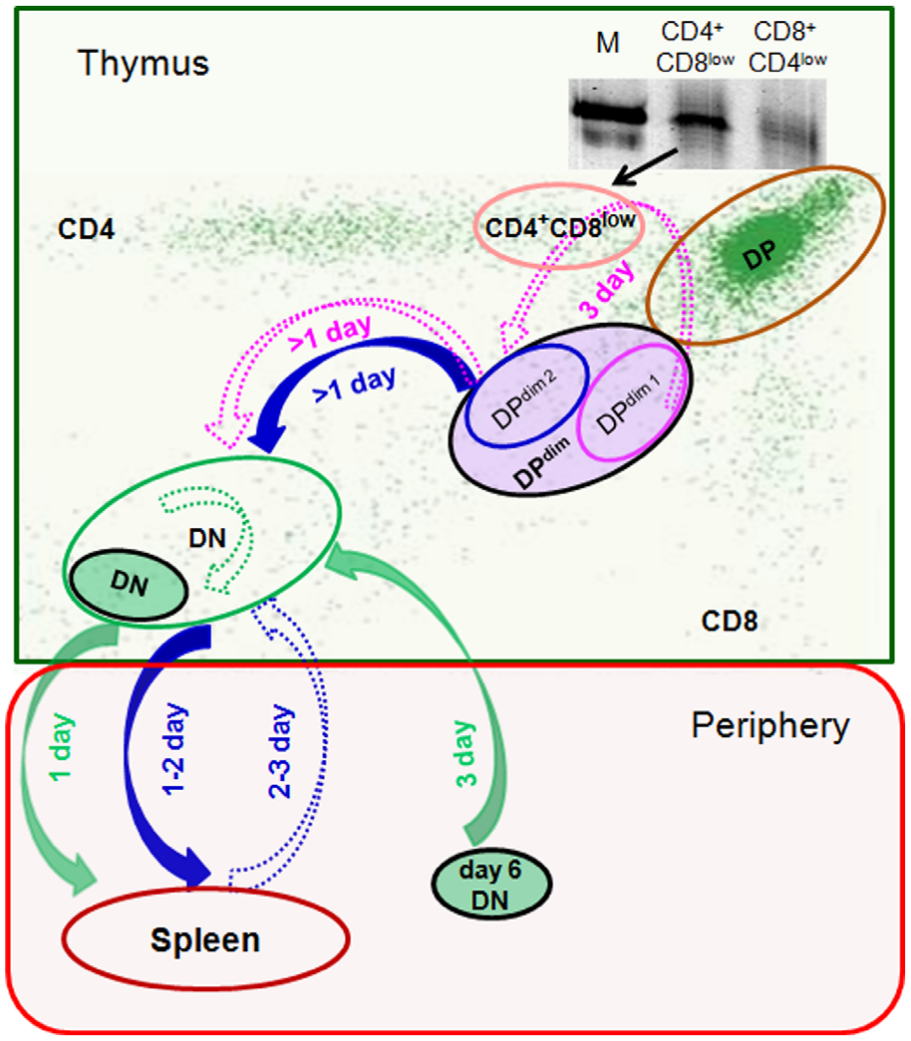
A model for the developmental dynamics of DN iNKT cells. The green box represents the thymus, and the red box the periphery. Filled ovals outlined in black identify cells transferred in the different experiments. Filled arrows and the timing next to them show the experimental findings. Open ovals represents specific compartments. Open arrows and the timing next to them are based on our interpretation. The gel insert shows the iNKT rearrangement in CD4^+^CD8^low^ and CD8^+^CD4^low^ cells.

It is interesting that one day after injection of DP^dim^ cells, iNKT rearrangement was detected in DN thymocytes of all mice analyzed (first phase), but was not detected in any mice three days after injection. iNKT rearrangement was detected again in DN thymocytes on day 4 in 66% of mice (second phase), and on days 5, 6, and 7 in all mice. We interpret the first phase as iNKT precursors in DP^dim2^ thymocytes give rise to DN iNKT within a day and these cells leave the thymus for the periphery within 1 or 2 days (Figure 6, solid blue arrows). Injecting DN cells into the thymus (solid green circle) showed that they could be observed in the periphery within a day (solid green arrow). The rapid exit of newly generated iNKT cells from the thymus is in agreement with previous reports that FITC-labeled thymocytes that are CD1d-αGC^+^ cells can be observed in the spleen in 24 hours [9].

The second phase could formally be explained in three ways: 1) Generation of iNKT cells from DP^dim1^ thymocytes, (Figure 6, dotted purple arrows). 2) Return of peripheral iNKT cells back to the thymus (dotted blue arrow). 3) Expansion of a very small number of DN iNKT cells in the thymus (dotted green arrow).

The second phase of DN iNKT cell observation could clearly come from the maturation of DP^dim1^ cells that had matured to DP and then became DP^dim2^ cells (Figure 6, dotted purple arrows). The lack of iNKT in DN thymocytes three days after injection and their appearance at day four indicates that four days would be required to mature from DP^dim1^ to DN. Since 18 hr or less is needed to move from DP^dim2^ to DN (first filled blue arrow), we assume that the same is true for DP^dim1^ that have matured to DP^dim2^ (second dotted purple arrow). Therefore, approximately three days (first dotted purple arrow) are needed to mature from DP^dim1^ to DP^dim2^. The 3 day time is in keeping with the 3.5 days that was reported for CD3^−^CD4^−^CD8^+^ (immature CD8) to become CD3^+^DP and CD3^+^CD4^+^) [19].

We draw the maturation pathway between the two DP^dim^ populations to include the results of experiments by Benlagha and colleagues who showed that injection of CD4 positive CD1-tetramer binding cells into thymi results in DN iNKT [5]. Because of previous reports that CD4^+^CD8^low^ populations are involved in DP to SP thymocyte maturation [23], we examined this population and found cells with the iNKT rearrangement (Figure 6, gel insert). This was not the case for the CD8^+^CD4^low^ cells. Thus we propose that after α-chain rearrangement has taken place in DP cells, iNKT start to down-regulate CD8 prior to down-regulating CD4. The opposite, down-regulating CD4 first, does not appear to be common.

The second phase cannot be due only to DP^dim1^ maturation, as it would be transitory since the newly arrived day 4 DN would be gone by day 6. The detection of DN iNKT cells at days 6, 7, and later argues that another mechanism is also contributing.

Long term observation of DN iNKT in the thymus could be due to return from the periphery (Figure 6, dotted blue line). Two lines of evidence, one direct and one indirect support this possibility. Peripheral injection of DN thymocytes that themselves were derived from DP^dim^ (filled green circle, day 6 DN) showed that these cells can return to the thymus (solid green line) in three days. The decrease in the percentage of animals that had iNKT cells in the spleen at day 5 compared with day 4 (Figure 2B) is compatible with the migration of iNKT cells from the spleen to other tissues that could include the thymus.

This interpretation appears to differ from the previous study of Berzin et al [14] that found no evidence for iNKT cells recirculating back to the thymus from periphery. However, their study focused on the predominant CD4^+^ iNKT population and these may differ in their characteristics from those iNKT that become DN. The role of CD4 in selection of iNKT and its retention by a large subset of iNKT has not been well investigated. It has been reported that CD4 can interact with CD1d [24]. This interaction is needed for cytokine release by CD4^+^ iNKT, and does not involve class II MHC [25]. DN iNKT recognition of CD1d loaded with lipid may be a simpler process.

Another possible mechanism that could contribute to the second phase would be the extensive proliferation of a small subset of DN iNKT cells that are retained in the thymus and whose level was below our measurement detection limit at day 3 (Figure 6, dotted green arrow). This subset would have to expand greatly to be detected on day 4. We have observed no evidence for a blasting population in day six post-transfer DN CD44^+^ cells as compared to zero day controls (data not shown).

Most likely, the second phase represents the generation of a steady state pool of thymic resident DN iNKT predominantly composed of cells that have matured in the periphery and returned to the thymus. As long as iNKT production is maintained there will also be a subset of cells representing DN iNKT generated from DP^dim1^ cells that represent a transient immature population that is on its way to the periphery.

We also show that early iNKT maturation from DP^dim^ cells includes rapid upregulation of CD44 and down regulation of CD4 and CD8. CD25 down regulation takes longer. Because day 6 represents maturation of DP^dim1^ and return of peripheral iNKT cells we can not determine the exact time of CD25 loss. However by day 28 the matured iNKT have lost.CD25 and their CD44 CD25 expression pattern resembles that of the steady state.

In summary, we propose a model (Figure 6) for the dynamics of selected iNKT cells in DP^dim^ in which it takes the immediate iNKT precursors in DP^dim^ (i.e. DP^dim2^) less than 1 day (18 hours) to lose the remaining CD4 and CD8 expression and appear as DN iNKT cells. These DN cells leave the thymus in 2–3 days and are observed in the spleen. iNKT thymic residents are generated predominantly from the relatively rapid return of mature peripheral iNKT cells back to the thymus (day 2–3 post exit). Overall, the dynamics of iNKT cell maturation from DP^dim^ to exit from the thymus are very rapid. A similar time frame has been reported for conventional T cells in which naïve αβ T cell precursors exit the thymus in 4–5 days [26]. Our study is another example of measuring maturation in the thymus and showing that it is a very rapid process.

## Materials and Methods

### Mice

C57BL/6 (6–8 weeks old) acted as cell donors, and TCRα^−/−^ (4–6 weeks old) as recipients. TCRα^−/−^ mice cannot express the TCRα chain because exon 3 of the TCRα constant gene is interrupted. All mice were obtained from Jackson Labs (Bar Harbor, ME), and were housed under controlled pathogen-free conditions. All animal procedures followed national and institutional guidelines and were approved by the Animal Care and Use Committee of the Medical College of Wisconsin; Protocol # 220-03-1: In Vivo Thymocyte Injection into the Murine Thymus.

### Antibodies and Reagents

CD4 (Clone CT-CD4)-Tri-color, CD8α (Clone: CT-CD8α)-PE and Tri-color, CD3-e (Clone: 500-A2)-FITC and Tri-color. CD25 (Clone: PC61 5.3)-R-PE, and CD44 (Clone: IM7.8.1)-FITC. All antibodies were purchased from Caltag Laboratories (San Francisco, CA).

### Primers

Poly T primer 5′TTTTTTTTTTTTTTTTTT

Cβ dir, 5′CCACCCAAGGTCTCCTTGTTTGAGCC 3′

Cβ anti, 5′-/56-FAM/GCGGAAGTGGTTGCGAGGATTGTGCC3′

Vα14, 5′GTGTCCCTGACAGTCCTGGTT3′

Jα18, 5′-/56-FAM/CAGGTATGACAATCAGCTGAGTCC3′

All primers were synthesized by Integrated DNA Technologies (Coralville, Iowa).

### Single Cell Suspension and Enrichment

Mice were euthanized, and spleen and thymi were removed. Cell suspensions were prepared by gently pressing the tissues with a 3 ml syringe plunger through a 70 um pore size screen using PBS solution containing 2% FCS. Splenocytes were treated with red cell lysis buffer, and B cells were removed using Miltenyi CD19 Micro Beads and LD separation columns (Miltenyi Biotech, Auburn, CA) according to the manufacturer’s instructions. DN thymocytes enrichment was performed by depleting CD4 and CD8 expressing thymocytes using Miltenyi CD4 and CD8 Micro Beads and LD separation columns.

### Sorting of DP^dim^ Thymocytes

Total thymocytes from one to two C57BL/6 mice were stained with CD4 and CD8 antibodies, and DP^dim^ thymocytes were sorted using FACStar or FACSVantage (Becton Dickenson, San Jose, CA). The collection tube contained 0.5 ml fetal calf serum, and on average 677,000 DP^dim^ cells were collected from the thymus of a C57BL/6 mouse. The sorted cells were spun down and resuspended in PBS for injection into the thymi of TCRα^−/−^ mice. In one experiment, total thymocytes were stained for CD4, CD8, and CD44 antibodies, and CD44 positive (0.108×10^6^) and CD44 negative (0.804×10^6^) DP^dim^ thymocytes were collected.

### Intrathymic Injection

A mid-line incision in the upper thoracic region was made to expose the supra-sternal notch. A small (approximately 0.5 cm) longitudinal incision in the musculature above the sternum exposed the thymic lobes, and 12.5 µl of cell suspension (0.4×10^5^ to 1.5×10^5^ cells) were injected into each thymus lobe. The wound was closed using 5–0 nylon suture. All manipulations were performed in a sterile manner, and post-operative recovery was better than 96%. Animals were euthanized at different days after the injection. The time referred to as day 1 represents 18 hour.

### Intravenous Jugular Vein Injection

25 µl of cell suspension (0.88×10^6^ cells) were injected into the jugular vein. All manipulations were performed in a sterile manner, and post-operative recovery was better than 96%. Animals were euthanized at different days after the injection. The time referred to as day 1 represents 18 hour.

### Sorting of DN Thymocytes

Total thymocytes from TCRα^−/−^ mice at different days post DP^dim^ injection were enriched for DN thymocytes. The enriched population was stained with CD4 and CD8 antibody and sorted for DN thymocytes. On average, 1.4 million total DN thymocytes were collected from each TCRα^−/−^ mouse on different days post thymic injection of DP^dim^ thymocytes.

In two experiments, total thymocytes from two to three TCRα^−/−^ mice post DP^dim^ injection were pooled, enriched for DN thymocytes, stained with CD4, CD8, CD44, and CD25 antibodies, and four different populations of DN were collected based on CD44 and CD25 expression.

### DNA Preparation

DNA from the sorted thymocytes and B cell depleted splenocytes was prepared by incubating the cells in acid lysis buffer (10 mM Tris, 0.4 M NaCl, 2 mM EDTA, pH 8.2) in the presence of 0.4% SDS and 0.4 mg/ml Proteinase K in a 42°C water bath overnight. Proteins were removed by adding NaCl (final concentration 1.3 M), followed by centrifugation. DNA was precipitated from the supernatant by adding cold pure ethanol (two times the volume). The DNA pellet was washed with cold 75% ethanol and resuspended in filtered distilled water, and the concentration of the DNA solution was measured by UV spectrophotometer.

### DNA Titration

Different amounts of DNA (20, 40, 80, and 160 ng) were used for amplification of TCR beta constant (TCRCβ) using Cβ direct and Cβ anti primer pairs. Amplification was performed for 24 cycles. A description of the PCR conditions has been provided in [27].

### Quantification of PCR Products

Equal volumes of the labeled PCR products were analyzed on denaturing polyacrylamide gels and scanned using a FluorImager (Molecular Dynamics, Sunnyvale, CA). Data was collected as a 16-bit Tiff file. Band intensities were further analyzed using Image Quant software.

### Assessment of iNKT by Vα14-Jα18 Rearrangement

To measure the canonical Vα14-Jα18 iNKT rearrangement, titrated amounts of DNA were amplified using Vα14-specific and Jα18-specific primers (Figure S3). The single band in the DN lane represents the canonical Vα14-Jα18 rearrangement (selected iNKT precursors). In contrast, the multiple bands in the DP lane represent different Vα14-Jα18 rearrangements. It should be noted that the DP thymocytes show a single base pair (BP) spacing due to chromosomes with out-of-frame rearrangements or the retention of excision circles with out-of-frame rearrangements. In analyzing samples for iNKT rearrangements, titrated amounts of DNA were used for amplification of Vα14 and Jα18. Thirty-two amplification cycles were used for the analyses of wild-type mice and 35–37 cycles for experiments using TCRα chain null mice.

### Sequencing

The Vα14-Jα18 PCR product was purified, and the DNA was used for sequencing using Vα14 primer and ABI Prism Big Dye Terminator Cycle Sequencing Kit (Applied Biosystems, Foster City, CA).

### Statistical Analysis

Fisher’s exact test is used to compute the P value from 2×2 contingency tables, and Zar’s method is used for comparison of the regression lines.

## Supporting Information

Figure S1 **Example of iNKT rearrangement analysis for DN thymocytes and total splenocytes from TCRα^−/−^ mice injected intrathymically with DP^dim^ cells.** A fluorogram of a gel analysis of five TCRα^−/−^ mice injected aliquots of the same DP^dim^ cell preparation. The days post DP^dim^ injection is shown above the lanes. “M” represents iNKT rearrangement in spleen of C57BL/6 and serves as a marker. “C” is the iNKT rearrangement in spleen of an uninjected control TCRα^−/−^ mouse. “B” represents the H_2_O amplification blank and serves as control for PCR reagents.
(TIF)

Figure S2 **Nucleotide sequence of the PCR product representing iNKT rearrangements.** A. Gel image of Vα14-Jα18 PCR product of DN thymocytes (left), and total splenocytes from TCRα^−/−^ mice 4days post DP^dim^ injection. B. DNA sequence fluorogram of the PCR products in A (reverse sequence).
(TIF)

Figure S3 **Vα14-Jα18 as a molecular marker for selected and expanded iNKT cells.** Vα14-Jα18 rearrangement in CD44^+^ CD25^−^ DN thymocytes that represent the stage after selection and expansion of iNKT cells (DN lane), and in DP thymocytes that represent the stage before expansion of iNKT cells (DP lane).
(TIF)
