# Supplementary figures and images for: Developmental Dynamics of Post-Selection Thymic DN iNKT
Source: PLoS One. 2012 Aug 22;7(8):e43509. doi: 10.1371/journal.pone.0043509 (PMC3425480; doi:10.1371/journal.pone.0043509)

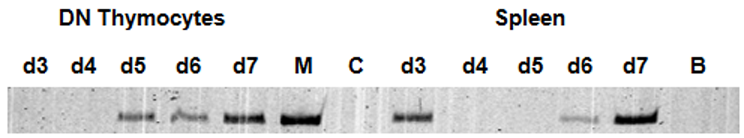

Supplement: Figure S1 — Example of iNKT rearrangement analysis for DN thymocytes and total splenocytes from TCRα−/− mice injected intrathymically with DPdim cells. A fluorogram of a gel analysis of five TCRα−/− mice injected aliquots of the same DPdim cell preparation. The days post DPdim injection is shown above the lanes. “M” represents iNKT rearrangement in spleen of C57BL/6 and serves as a marker. “C” is the iNKT rearrangement in spleen of an uninjected control TCRα−/− mouse. “B” represents the H2O amplification blank and serves as control for PCR reagents. (TIF) [file pone.0043509.s001.tif]

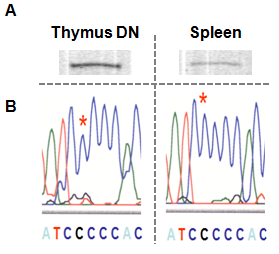

Supplement: Figure S2 — Nucleotide sequence of the PCR product representing iNKT rearrangements. A. Gel image of Vα14-Jα18 PCR product of DN thymocytes (left), and total splenocytes from TCRα−/− mice 4days post DPdim injection. B. DNA sequence fluorogram of the PCR products in A (reverse sequence). (TIF) [file pone.0043509.s002.tif]

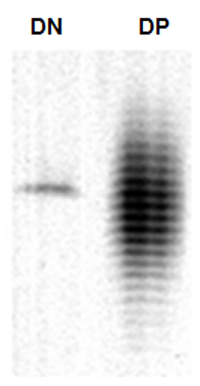

Supplement: Figure S3 — Vα14-Jα18 as a molecular marker for selected and expanded iNKT cells. Vα14-Jα18 rearrangement in CD44+ CD25− DN thymocytes that represent the stage after selection and expansion of iNKT cells (DN lane), and in DP thymocytes that represent the stage before expansion of iNKT cells (DP lane). (TIF) [file pone.0043509.s003.tif]
